# Optical and Electrical Characteristics of Silicon Nanowires Prepared by Electroless Etching

**DOI:** 10.1186/s11671-017-2197-3

**Published:** 2017-06-24

**Authors:** Sabar D. Hutagalung, Mohammed M. Fadhali, Raed A. Areshi, Fui D. Tan

**Affiliations:** 1 Physics Department, Faculty of Science, Jazan University, Jazan, Saudi Arabia; 2 Physics Department, Faculty of Science, Ibb University, Ibb, Yemen; 3 School of Materials and Mineral Resources Engineering, Universiti Sains Malaysia, Penang, Malaysia

**Keywords:** Silicon nanowires, Electroless etching, Microstructures, Reflectance, Band gap energy, Resistivity

## Abstract

Silicon nanowires (SiNWs) were fabricated by the electroless etching of an n-type Si (100) wafer in HF/AgNO_3_. Vertically aligned and high-density SiNWs are formed on the Si substrates. Various shapes of SiNWs are observed, including round, rectangular, and triangular. The recorded maximum reflectance of the SiNWs is approximately 19.2%, which is much lower than that of the Si substrate (65.1%). The minimum reflectance of the SiNWs is approximately 3.5% in the near UV region and 9.8% in the visible to near IR regions. The calculated band gap energy of the SiNWs is found to be slightly higher than that of the Si substrate. The *I*–*V* characteristics of a freestanding SiNW show a linear ohmic behavior for a forward bias up to 2.0 V. The average resistivity of a SiNW is approximately 33.94 Ω cm.

## Background

The physical properties of nanomaterials are significantly different from those of their bulk counterparts and primarily depend on their size and shape. For example, bulk silicon emits a weak infrared photoluminescence because it possesses an indirect band gap [1]. This indirect band gap prevents efficient interband radiative recombination. Therefore, one-dimensional (1D) structures, such as silicon nanowires (SiNWs), are introduced, as they are able to emit efficiently through photoexcitation due to quantum size effects. In the confined dimensions of nanomaterials, the limited motion of electrons contributes to an enhancement in the electrical properties of their devices [2].

The formation of 1D Si nanostructures provides novel device properties, such as efficient charge transport and controllable optical properties. SiNWs are promising candidates for future nanoelectronics and especially optoelectronic devices due to their remarkable electrical, optoelectronic, and mechanical properties [3, 4]. Hence, a large amount of research has been focused on the utilization of SiNWs in a wide range of applications [5].

There are numerous methods to fabricate SiNWs, including both top–down and bottom–up approaches. However, all of these methods have their own advantages and disadvantages. Among the various methods, the most common method is a vapor–liquid–solid (VLS) growth mechanism, which was first proposed by Wagner in 1960s during his studies of large single-crystalline whisker growth [6]. Since then, many researchers have fabricated SiNWs using chemical vapor deposition (CVD) [7, 8], laser ablation [9], thermal evaporation [10], and molecular beam epitaxy [11]. However, these bottom-up techniques usually require complex equipment, high temperature, high vacuum, and hazardous silicon precursors, all of which drastically increase the cost of the processes. Moreover, SiNW production over large areas is not possible due to limitations in the growth setups used [12]. Therefore, a simple method, namely, the electroless etching method, has been proposed as a promising synthetic method. The first introduction of metal-assisted chemical etching of Si was reported in 1997, where porous Si was fabricated by etching an aluminum (Al)-coated Si substrate in a solution composed of hydrofluoric acid (HF), HNO_3_, and H_2_O [13]. Since then, many researchers have employed similar methods to fabricate SiNW arrays using various etching solution systems, such as HF/AgNO_3_ or HF/H_2_O_2_/AgNO_3_ [5, 12, 14–20].

The first part of this work focuses on the fabrication of SiNWs with desired structures, such as well-aligned, high-density, and high-uniformity structures in terms of size, length, and distribution. SiNWs were fabricated by the electroless etching of a Si wafer in a mixed solution of hydrofluoric acid and silver nitrate (HF/AgNO_3_ system). During etching of the silicon wafer, a selective area is etched away while the rest is preserved, and hence, vertically aligned wire structures in nanoscale range are formed. After etching, the silver precipitate on the SiNWs must be removed completely before proceeding to the next processes. This is because the silver precipitate may act as a barrier and influence the properties (especially the optical and electrical properties) of the SiNWs. Finally, the fabricated SiNWs were characterized for their microstructure, elemental composition, morphology, and optical and electrical properties.

Although many researchers have investigated the optical properties of SiNWs, most of them use transmittance measurements to determine the band gap energy. In this work, the optical properties of the SiNWs fabricated by electroless etching in HF/AgNO_3_ were determined from reflectance measurements. Additionally, the electrical characteristics of the SiNWs were investigated using conductive atomic force microscopy (AFM) measurements.

## Methods

### Fabrication of SiNWs

The SiNWs were synthesized by the metal-assisted electroless etching method using phosphorus-doped silicon wafers with a (100) orientation or n-type Si (100) wafers. The resistivity of the wafers ranged from 0.75 to 1.25 Ω cm, with thicknesses of 500–550 μm. The Si wafers were supplied by Siltronix Silicon Technologies, France. The wafers were then sequentially cleaned ultrasonically in acetone, ethanol, deionized water, and boiling piranha solution (H_2_SO_4_:H_2_O_2_ = 4:1; *v*/*v*) for 30 min. The cleaned wafers were rinsed with deionized water and lastly dipped into HF solution for 20 s followed by washing with deionized water to remove native oxides.

The bath solution for the electroless etching process was prepared by mixing a 5 M HF solution with a 0.01 M AgNO_3_ solution. HF was supplied by JT Baker, with a purity of 48%, CMOS grade. In addition, AgNO_3_ was supplied by QREC, with a purity of over 99%.

The pre-cleaned Si substrates were immersed into the prepared etchant solution (metal-assisted electroless etching). The etching temperature was 60 °C, and the etching time was 60 min [21]. The etching process was performed in a sealed Teflon vessel inside an HF fume cupboard. After the etching, the Si substrates were quickly rinsed several times with deionized water, followed by ultrasonic cleaning for 15 min in 3 mol/L *aqua regia* solution to remove silver deposit from the sample. Aqua regia solution is a mixture of nitric acid (HNO_3_) and hydrochloric acid (HCl) in a 1:3 volume ratio. The samples were rinsed again with deionized water and dried. HNO_3_, with a purity of 65%, was supplied by LABSCAN, and CMOS-grade HCl was supplied by MERCK, with a concentration of 12 mol/L.

### Characterizations

The microstructure, morphology, and chemical composition of the samples were characterized by field-emission scanning electron microscopy (FESEM) equipped with an energy dispersive X-ray (EDX) spectrometer (Zeiss Supra 35 VP) and transmission electron microscope (TEM) (Philips CM12). A PerkinElmer Lambda 35 Ultraviolet-visible spectrophotometer was used to investigate the reflectance in the range of 200–1100 nm. The surface topography and electrical characteristics of the SiNWs were investigated by AFM using a commercial Seiko SPI 3800N Series with a SPA-300HV microscope [22, 23]. *I*–*V* measurements at the selected points were performed by AFM in contact mode using a conductive AFM probe tip (gold-coated, ∅_TIP_ ≈ 20 nm) by applying a forward bias voltage ranging from 0 to 2 V to the AFM tip.

## Results and Discussion

### Microstructures

**Fig. 1:**
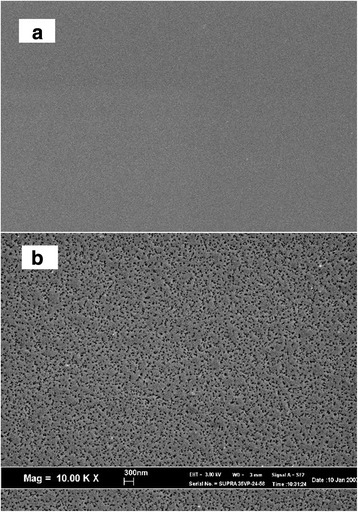
SEM images of **a** the pre-cleaned Si wafer and **b** the Si wafer etched in 5 M HF without AgNO_3_ solution

Figure 1 shows the SEM images of (a) the pre-cleaned n-Si (100) substrate before etching and (b) the n-Si(100) substrate etched at 60 °C for 60 min in 5 M HF. The bare silicon wafer possesses a smooth mirror-like surface free from contaminants (Fig. 1a). The formation of mesoporous structures was detected on the Si substrate etched in HF solution (Fig. 1b). HF is well known as an etchant solution for silicon oxide, and thus, the formation of pores on the Si surface may be due to the etching of its native oxide. However, the etching rate is very slow.

**Fig. 2:**
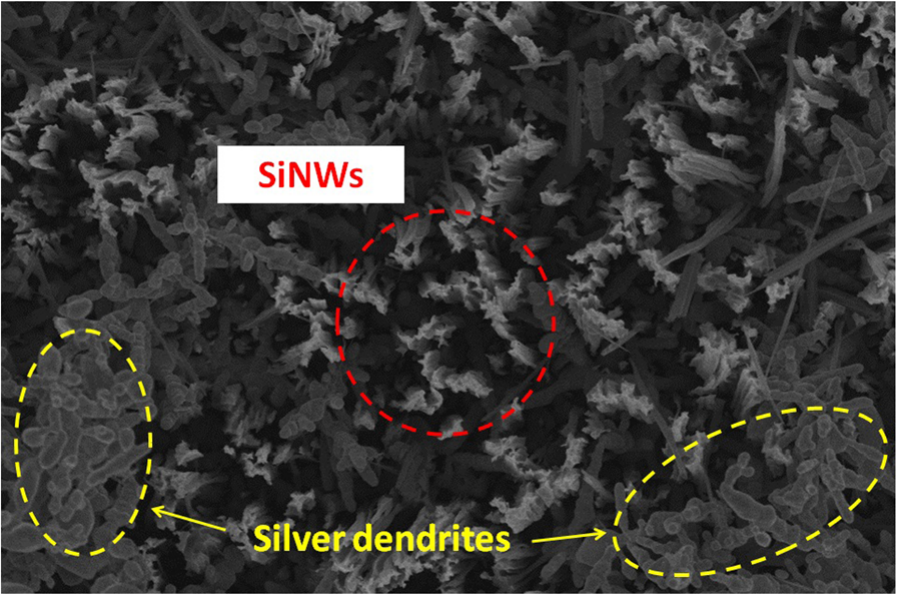
SEM image showing the formation of SiNWs with silver dendrites after the electroless etching process (before aqua regia cleaning)

The SiNWs were successfully formed by the silver-assisted electroless etching of a Si wafer in a solution containing 5 M HF and 0.01 M AgNO_3_ at 60 °C for 60 min. Figure 2 shows the SEM image of a substrate after the etching process. Vertically aligned SiNW arrays were formed on the substrate when silver ions were introduced into the etchant solution. In addition to nanowires, silver dendrites were also found on the SiNWs. They are either deposited on the tips of the wires or on the bottom of the substrate.

The formation of porous silicon in the early stage of SiNW fabrication in ionic metal etchant solution is initiated by hole generation. The hole acceptor is assumed to be the surface Si-H bond [24]. Silicon etching and silver deposition occur simultaneously at the Si wafer surface. The deposited silver atoms first form nuclei and then form nanoclusters distributed on the surface of the silicon wafer. These silver nanoclusters and the Si areas surrounding these silver nuclei could act as local cathodes and anodes, respectively, in the electrochemical redox reaction process, which can be formulated as two half-cell reactions:

$$ \mathrm{A}{\mathrm{g}}^{+} + {\mathrm{e}}^{-}\to \mathrm{A}\mathrm{g} $$

$$ \mathrm{S}\mathrm{i} + 6{\mathrm{F}}^{-}\to \mathrm{S}\mathrm{i}{{\mathrm{F}}_6}^{2-} + 4{\mathrm{e}}^{-} $$

Numerous nanoscale freestanding electrolytic cells were spontaneously assembled on the Si surface. During silver deposition, the silver nanoclusters, which act as cathodes, were successfully preserved, while the surrounding silicon, which acts as the anode, was etched away [12, 14, 25, 26].

**Fig. 3:**
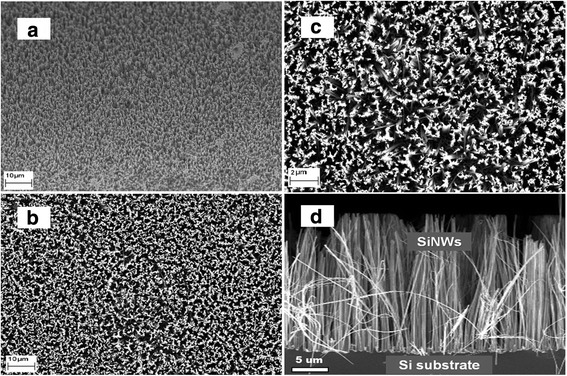
SEM images of SiNW arrays fabricated via the electroless etching method: **a** 45° view, **b**, **c** top view, **d** cross-section/side view

Figure 3 shows SEM micrographs of the SiNW arrays fabricated via the electroless etching method and after cleaning in aqua regia solution. High-density SiNW arrays can be observed from different views: 45° view (Fig. 3a), top view (Figs. 3b, c), and cross-section/side view (Fig. 3d). In addition, the SiNW arrays are all well aligned in the vertical direction, which follows the orientation of the silicon wafer substrate in the (100) direction. As shown in the cross-section view of the SiNWs in Fig. 3d, the average etching depth is approximately 20 μm, which corresponds to the length of the SiNWs, whereas the diameter is in the range of 20 to 300 nm.

A simple model for the formation of SiNWs during the silver metal-assisted chemical etching of Si was presented by Smith et al. [16]. When Si is immersed in HF/Ag^+^ solution, silver nanoparticles nucleate immediately and quickly grow. During the initial nucleation stage, individual hemispherical particles are essentially isolated on the Si surface and grow independently. Silver nucleation and growth is a highly dynamic process, allowing silver to dissolve, redeposit, and/or surface migrate to more energetically favored sites. Another comprehensive formation mechanism of porous SiNWs by the etching of Si wafers in HF/AgNO_3_ was proposed by Li et al. [18]. The porous structures within the SiNWs were attributed to Si oxidation induced by Ag nanoparticles. It was found that the higher HF concentration was favorable for the growth of SiNWs, and the formation of SiNWs was significantly affected by the Ag^+^ ion concentration.

Li et al. [17] also reported the fabrication of porous silicon nanowires (PSiNWs) using an etchant solution of HF/H_2_O_2_/AgNO_3_. In this system, the H_2_O_2_ species replaces Ag + as the oxidant, and the Ag nanoparticles function as catalysts during etching. A different morphology of SiNWs was found, in which the whole nanowire was covered by numerous porous structures. They found that the porosity could be controlled by adjusting the concentration of H_2_O_2_ [17].

In the past, it was speculated that metal-assisted etching is isotropic and that the noble metal always catalyzes etching along the vertical direction relative to the substrate surface [27–29]. Later, experiments revealed that etching was dominantly anisotropic, as non-vertical etching occurred for (111) and (110) substrates, resulting in slanted, aligned SiNWs [30–32]. This anisotropic etching along certain preferred crystallographic directions was ascribed to the back-bond breaking theory [33, 34]. During etching, it is necessary to break the back-bonds of the surface atom that connects to the underneath atoms for the oxidation and dissolution reaction on the surface to be activated. The number of back-bonds is determined by the crystallographic orientation of the substrate [29]. Each atom on the surface of the (100) substrate has two back-bonds, whereas on the (110) and (111) surfaces, each atom has three back-bonds. Hence, the fabrication of SiNWs on (100) substrates is easier than on the others [35].

### Elemental Composition

**Fig. 4:**
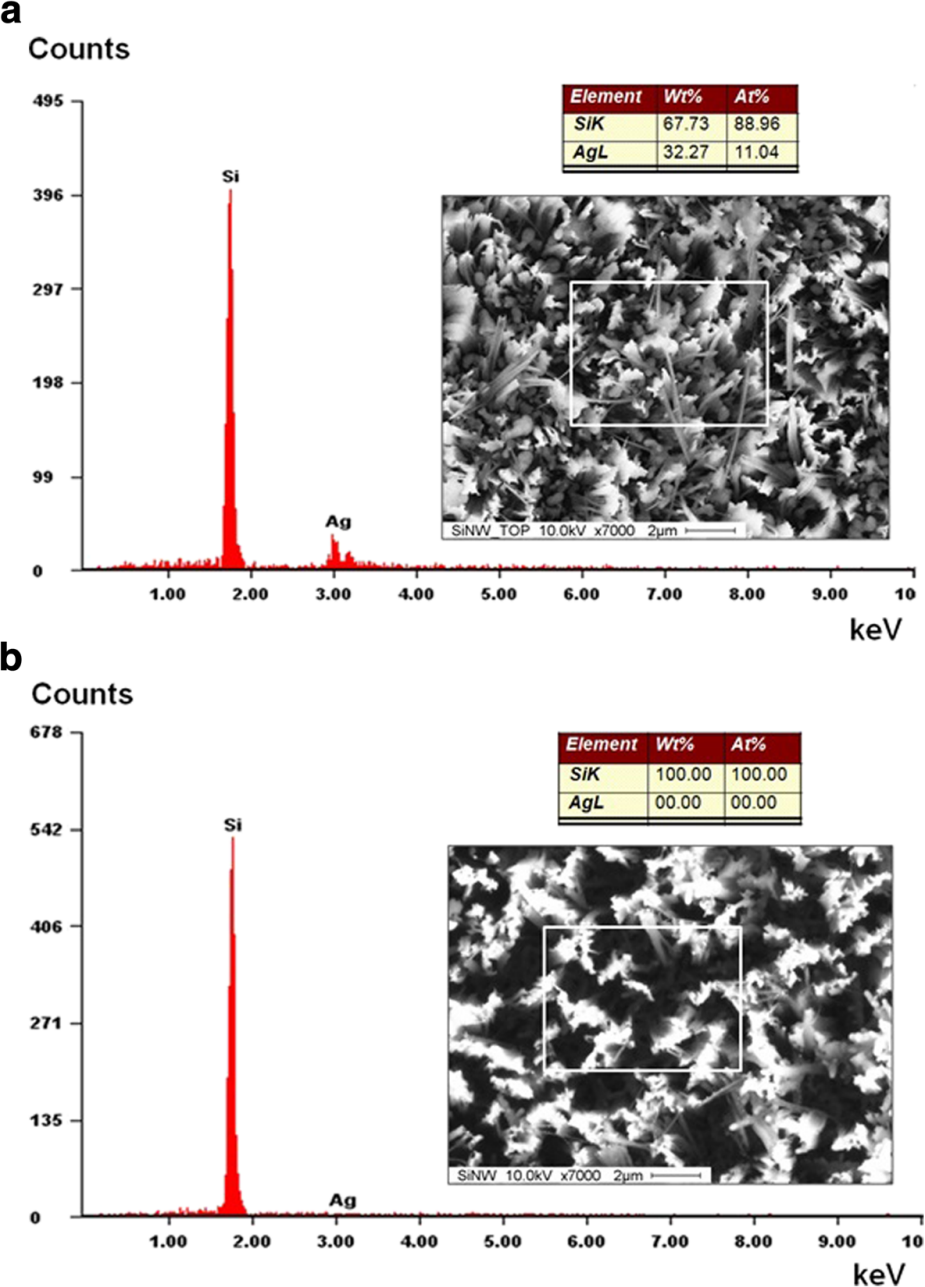
EDX analysis results of SiNW arrays after the etching processes: **a** before aqua regia cleaning (88.96 at.% Si and 11.04 at.% Ag) and **b** after aqua regia cleaning (100.00 at.% Si and 0.00 at.% Ag)

Figure 4 shows the EDX analysis results of the SiNW arrays before and after ultrasonic cleaning with aqua regia solution. The results show that the elements contained in the samples are mainly Si and Ag. Silver was clearly deposited at the wire tip as well as in the valley among the wires after the etching process. As shown in Fig. 4a, a large amount of silver (approximately 11.04 at% Ag) was detected. However, a purely Si composition (100 at% Si) was obtained after cleaning in aqua regia solution (Fig. 4b).

### Size and Shape of SiNWs

As previously reported, the most suitable concentration of etchant is 0.01 mol/L for AgNO_3_ and 5.0 mol/L for HF. However, one major concern of this method is that the formed silver nanoclusters tend to deposit randomly on the surface of the Si substrate. This phenomenon contributes to the difficulty in tailoring the uniformity, alignment, length, and diameter of the fabricated nanowires. Moreover, the silver nanoclusters deposited on the surface of the Si wafer were found to have various sizes and shapes, because the random paths to contact the selected seed allow the particles to diffuse and stick to the formed structure. This led to the formation of SiNW arrays with non-uniform size and shape [5, 12, 15–20, 28, 36–38].

In general, the morphologies of the electroless-etched Si structures are defined by the shape of the Ag catalyst deposits. Well-separated Ag particles usually result in well-defined pores, whereas etched structures may evolve from the pores into wall-like or wire-like structures when Ag particles are deposited in bundles [29]. In other words, the catalytic activity of the Ag ion is determined by the exposed facets. Thus, it is reasonable that the nanowire structure produced neither a single individual wire nor a wall-like structure but a mixture of both.

**Fig. 5:**
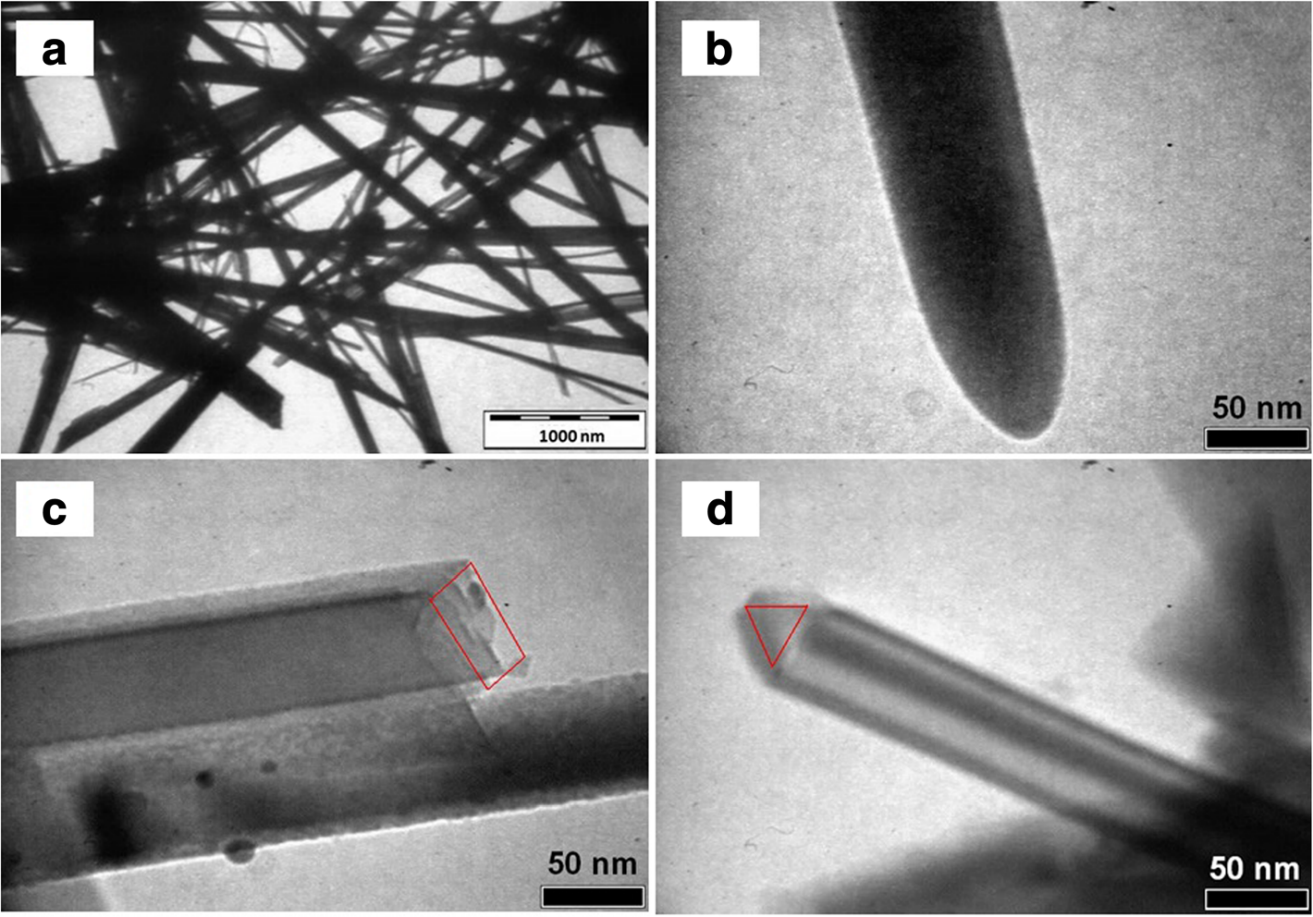
TEM images of loose SiNWs: **a** bundle of SiNWs with various sizes and shapes, **b** round-shaped SiNW, **c** rectangular-shaped SiNW, and **d** triangular-shaped SiNW

Figure 5 shows the morphology of the SiNWs. TEM analysis was performed on SiNWs pre-removed from the substrate. The results show that the size (diameter) of the SiNWs is in the range of 20 to 200 nm (Fig. 5a). Various shapes of SiNWs were found, including round shapes (Fig. 5b), rectangular shapes (Fig. 5c), and triangle shapes (Fig. 5d). The shape variation of the SiNW products are due to the inhomogeneous formation of silver deposits on the Si substrate surface. The size and shape of the SiNWs could be well controlled by controlling the geometry shape, size, and positions of the local cathodes (silver nanoclusters). This can be achieved via electroless etching using a suitable template, such as a monolayer of polystyrene spheres [39]. The results are similar to the formation of ordered honeycomb structures during anodization [40, 41].

### Optical Properties

The fabrication of SiNWs is a surface modification technique that aims to minimize the reflection of incident light and increase the absorption as much as possible. From the observations, SiNWs on the Si substrate are black in appearance compared to the bare Si wafer, which has a shiny surface. The antireflective nature of the SiNWs has drawn attention since one of the major energy loss mechanisms of solar cells is optical reflection; the utilization of these nanostructures in photosensitive devices may eliminate the need for antireflective coatings [5, 19, 20, 28]. To quantify the optical properties of the fabricated SiNWs, an ultraviolet-visible (UV-Vis) spectrophotometer was used to measure the reflectance of the samples.

**Fig. 6:**
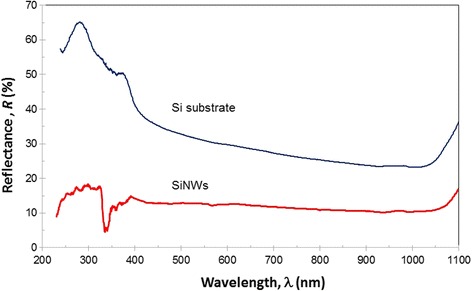
Reflectance (*R*) versus wavelength (*λ*) for a Si substrate and the SiNW arrays

Figure 6 shows the variation in reflectance (*R*) depending on the wavelength (*λ*) of incident radiation on the Si substrate and SiNW arrays. The measurement was obtained by illuminating samples with radiation varying from the ultraviolet region (UV) to the infrared region (IR) with wavelengths ranging from 200 to 1100 nm. It is clearly seen that the reflectance of the SiNWs is much lower than that of their bare Si wafer counterpart.

Referring to Fig. 6, the reflectance spectrum of the SiNW arrays seems rather stable (almost flat curve) in the visible to near IR regions (400–1100 nm). This simply indicates that the SiNW arrays are very sensitive to visible and near IR radiation. This wavelength range (400–1100 nm) is essential for photosensitive device applications. The average reflectance of the SiNW arrays is approximately 12% in the visible region and 10% in the IR region. However, the Si substrate gave a much higher average reflectance. The maximum reflectance of the Si substrate is 65.1%, which is much higher than the maximum reflectance of the SiNWs (19.2%) in the same wavelength range (near UV region). Additionally, the minimum reflectance of the SiNWs is approximately 3.5% in the near UV region and 9.8% in the visible to near IR regions. A detailed comparison of the reflectance in the UV, visible, and IR regions is summarized in Table 1. The results reveal that modifying the surface morphology of the Si substrate to form SiNWs successfully reduced the reflection loss of incident radiation over a broad spectral range. This property meets the requirements for the application of SiNWs in photosensitive devices able to effectively detect incident light.

**Table 1:** Reflectance maximum (R-max) and minimum (R-min) of the Si wafer and SiNWs in the UV, visible, and IR regions

Sample	UV region (200–400 nm)		Visible region (400–750 nm)		IR region (750–1000 nm)	
	R-max (%)	R-min (%)	R-max (%)	R-min (%)	R-max (%)	R-min (%)
Si substrate	65.1	38.8	38.8	25.5	25.5	22.1
SiNWs	19.2	3.5	15.1	12.1	12.1	9.8

The results are in agreement with the reflectance results previously reported [19, 20, 42]. Jia et al. [42] found that the reflectance of black Si (bare Si etched with different post-etch treatments) is approximately 10% over the whole visible spectrum as well as in the near UV and near IR regions. Additionally, the reflectance of a polished wafer without anti-reflection coating varies between 30 and 50% over the same wavelength range [42]. Li et al. [19] reported that nanotextured multi-crystalline silicon (mc-Si) shows an outstanding anti-reflectance ability of approximately 5.6%. This result was obtained for a Si wafer etched in HF/H_2_O_2_/AgNO_3_ under optimized fabrication conditions. Moreover, the antireflection properties gradually increase with increasing uniformity of the nanowire arrays, which decreases when the nanowire length is too long, which leads to the agglomeration of nanowires. Similar or even better results have been reported for SiNWs treated with KOH to further reduce the reflectance loss. Under these conditions, large-scale SiNW arrays with the ultra-low antireflection ability of ~3.4% can be obtained [20].

Since decades ago, the reflection loss was monitored by using an antireflection coating. However, these coatings have resonant structures and work effectively only in a limited spectral range and for specific angles of incidence [28]. SiNWs are surface relief structures with dimensions smaller than the wavelength of the incident light. Wavelengths larger than the nanowire diameter pass through the sample and are only absorbed by multiple diffuse scattering. In the longer wavelength region, the minimized reflection is due to the high diffraction of light between the SiNWs [43]. These deep profiles can suppress the Fresnel reflection substantially over a wide spectral bandwidth [28]. In addition, the SiNW arrays demonstrate strong optical absorption due to light trapping effects and optical antenna effects, leading to suppressed optical reflection [44].

Many researchers have claimed that nanowires with different morphology than the bulk produce band gap broadening, which is attributed to the wide absorption spectrum and optical transmission range. By applying the Kubelka-Munk [K-M or F(R)] method [45], the band gap energy of the sample can be determined by the following equation:

$$ F(R)=\frac{\left(1- R\right)2\ }{2 R} $$

where *R* is the reflectance and F(R) is proportional to the extinction coefficient (*α*). In this work, [F(R)*hv]^*n*^ versus the photon energy (*hν*) for the Si substrate and SiNWs is plotted, where *h* is Planck’s constant (4.1357 × 10^−15^ eV s), *v* is the light frequency, and *n* is a coefficient associated with the electronic transition (2 for direct allowed transitions and 1/2 for indirect allowed transitions). By extending the tangential line of the gradient beyond the *x*-axis, the point at which the line intersects with the *x*-axis is the estimated band gap energy of the sample.

**Fig. 7:**
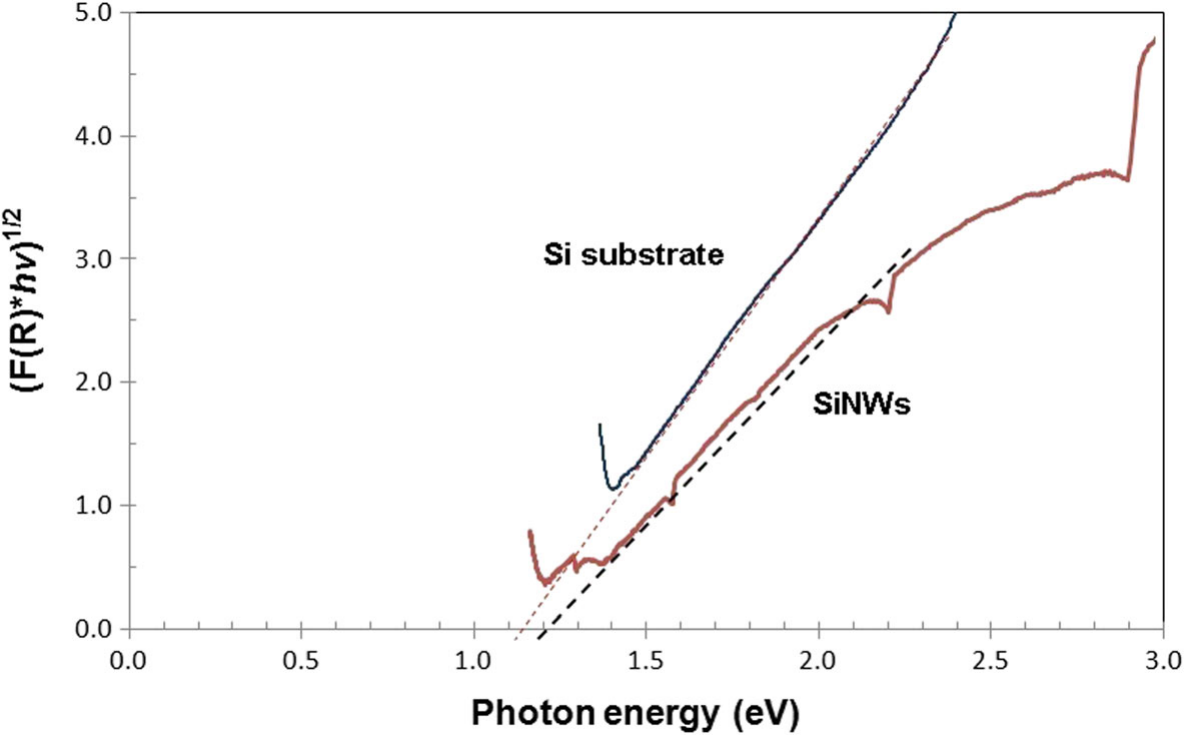
Plot of (F(R)*hν)^1/2^ versus photon energy (*hν*) for a Si substrate and the SiNW arrays

Figure 7 shows the plots of (F(R)*hν)^1/2^ versus photon energy (*hν*) for the Si substrate and SiNWs. It was found that the band gap energy of the SiNWs is slightly higher than that of their bare Si wafer counterparts. The calculated band gap energy, *E*_g_, is approximately 1.15 eV for the Si substrate and approximately 1.20 eV for the SiNWs. These results are in agreement with those of Kurokawa et al. [39]. This group reported the band gap of SiNW arrays to be approximately 1.2 eV, as obtained from cathodoluminescence measurements. The band gap widening phenomenon can be explained by the quantum confinement (QC) effect. According to QC theory, the band gap should increase with a size decrease of the nanostructure and lead to a blueshift [46].

Ng et al. [47] reported that the band gap width of the SiNWs increases with a decrease in the diameter of the nanowire structure. Moreover, the high surface area-to-volume ratio afforded the SiNWs with direct band gap behavior, which became more impressive as the wire diameter decreased [48]. Small-diameter Si wires exhibit a direct band gap. A band gap of over 2.5 eV was obtained for SiNWs with a diameter of approximately 1 nm, as determined by density functional theory calculations [48]. Li and Bohn [49] demonstrated the fabrication of light-emitting devices using porous Si prepared by the metal-assisted electroless etching method. They found that the large surface area led to a shift in the band gap as well as efficient luminescence properties of the porous nanostructures [49].

### Electrical Properties

Atomic force microscopy (AFM) was used to characterize the surface topography and electrical properties of the synthesized SiNWs. In AFM, a small probe is scanned across the sample, and information regarding the surface of the sample is gathered from the interaction of the probe with the surface. The results obtained are in the form of physical topography and measurements of the physical, magnetic, and chemical properties of the sample. AFM measurements are based on the deflection of the cantilever as the sample surface topography changes and the interatomic forces between the probe tip and the sample surface are varied.

**Fig. 8:**
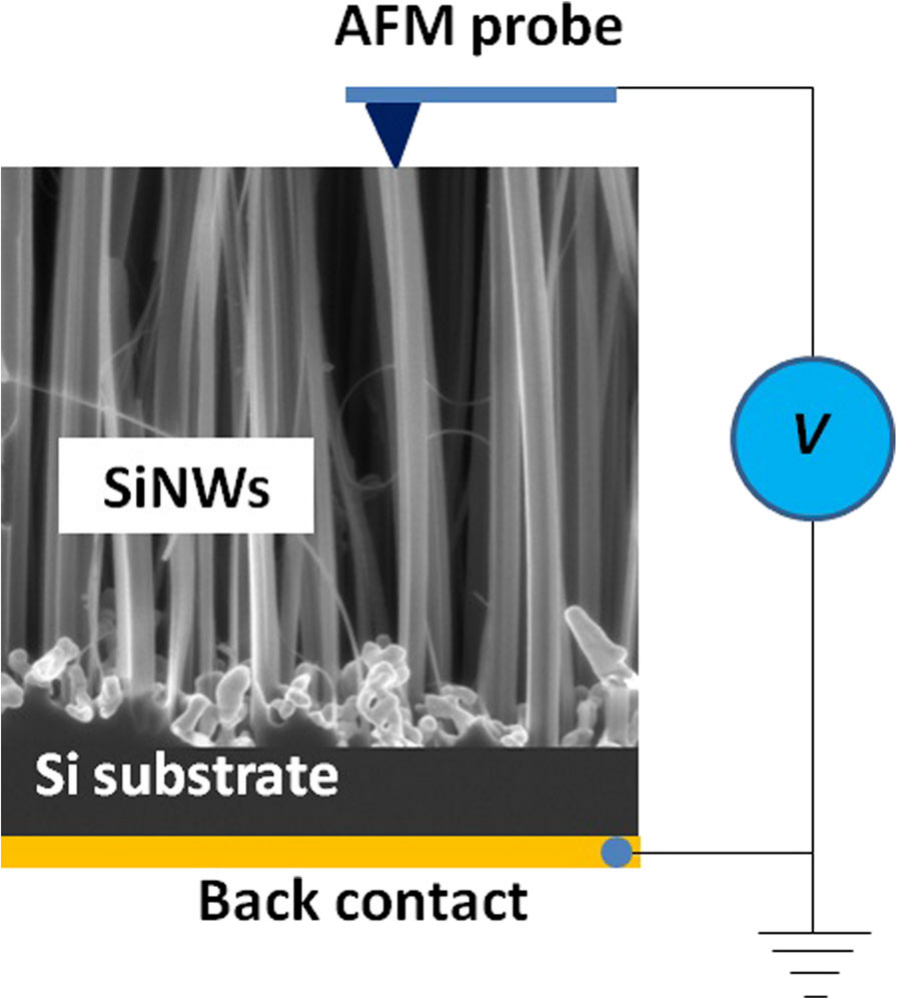
Schematic diagram of the experimental setup for the electrical measurement by AFM

Non-contact mode operation was used for surface topography imaging; however, contact-mode AFM was applied for the electrical measurements [22, 23, 50]. In this work, a commercially available conductive AFM probe was used to obtain the current versus voltage (*I*–*V*) characteristics and surface topography of the SiNW arrays. Figure 8 shows a schematic diagram of the experimental setup for *I*–*V* characterization. In this experiment, a conductive AFM probe (gold-coated tip, radius approximately 10 nm, force constant 40 Nm^−1^, and resonant frequency 300 kHz) was in contact with the top end of a SiNW via contact-mode AFM operation. A ramped voltage from 0 to 2 V.

**Fig. 9:**
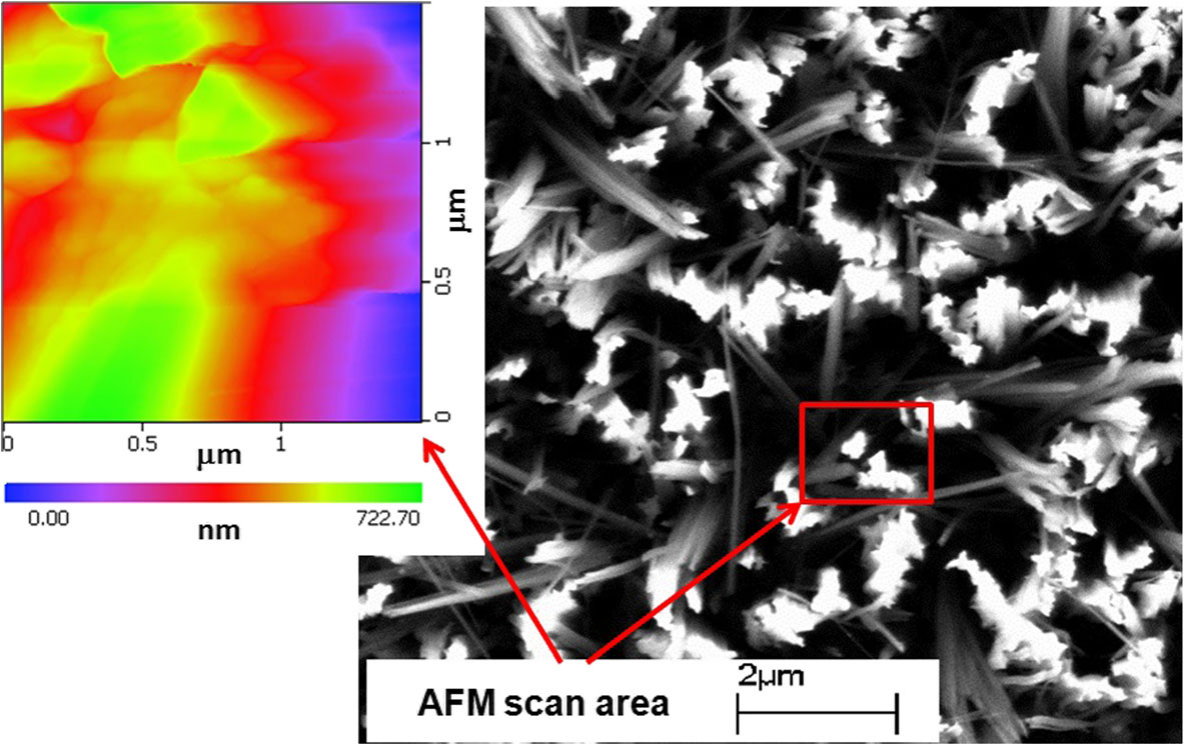
SEM and AFM images of the vertically aligned SiNWs. A triangle-shaped wire was chosen for the electrical measurement by AFM

Figure 9 shows a top-view SEM image and a 2D AFM topography image of the vertically aligned SiNW arrays over a scanning area of 1.5 μm × 1.5 μm. Individual as well as grouped nanowires can be observed. From the topography image, the peak-to-valley surface roughness was found to be 722.7 nm with an average roughness of 127.4 nm.

**Fig. 10:**
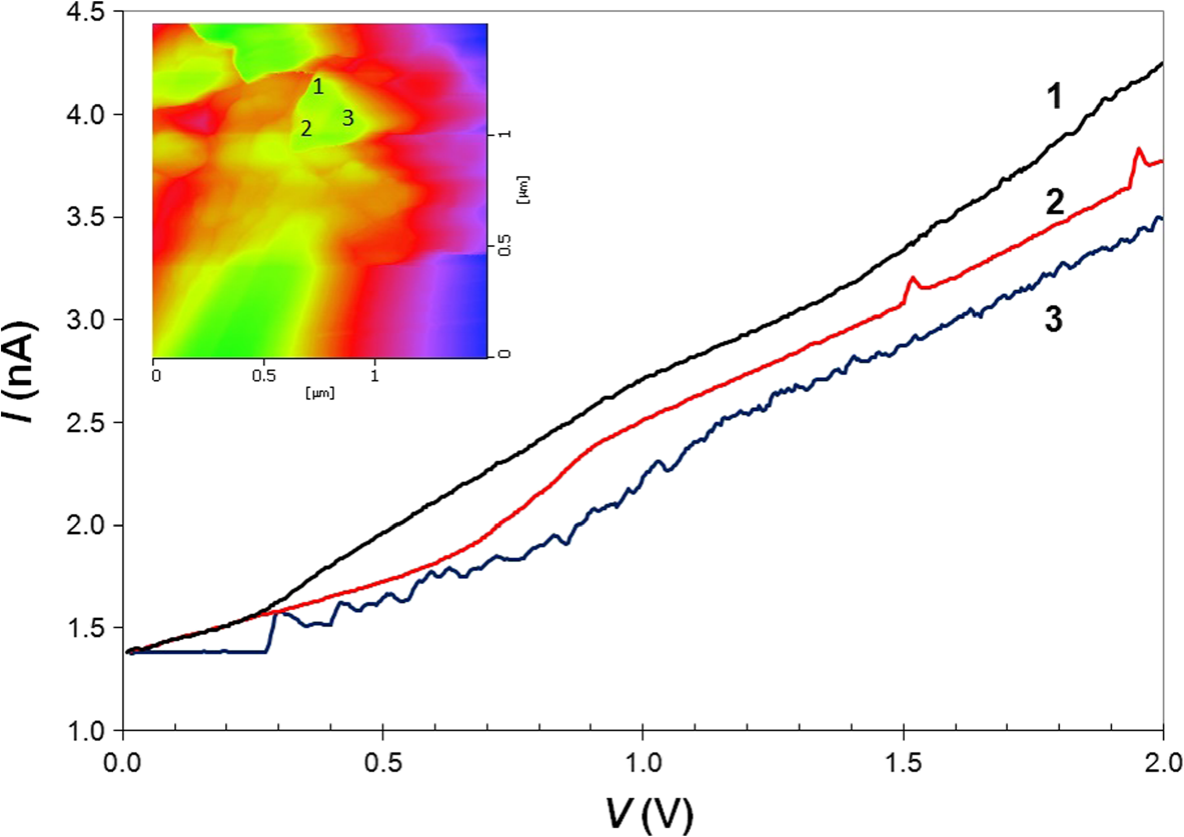
*I–V* curves of an individual freestanding SiNW measured by AFM. The characteristics correspond to resistor behavior. The *inset* shows the AFM scanning area and AFM probe positions for the electrical measurement

Figure 10 shows the *I*–*V* characteristic curves at various tip positions on the top of a freestanding SiNW. The characteristics correspond to a resistor, and the nanowire has a linear ohmic behavior at all the points for a forward bias up to 2.0 V. In this case, the etching depth (nanowire length) is approximately 722.7 nm, as estimated from the peak-to-valley surface roughness (see AFM image in Fig. 9). The electrical resistance can be determined from the slope of the *I*–*V* characteristic curves in Fig. 10.

The resistivity, *ρ*, can be obtained from Ohm’s law:

$$ \rho =\frac{RA}{L} $$

where *R* is the resistance, *A* is the contact area corresponding to the AFM tip area, and *L* is the length of the wire. Using the wire length (722.7 nm) from the AFM image and the apex radius of the AFM tip (approximately 10 nm), the average resistivity was found to be 33.94 Ω cm. For further details, see the calculated resistance and resistivity in Table 2. This result is much higher than the resistivity of SiNWs reported by Bauer et al., where they obtained a resistivity of 0.85 Ω cm for nanowires grown by molecular-beam epitaxy on an *n*+ silicon substrate [51].

**Table 2:** The resistance and resistivity of a single wire measured by AFM at different points

Point	*R* (GΩ)	*ρ* (Ω cm)
1	6.94	30.17
2	7.91	34.36
3	8.58	37.28
Average	7.54	33.94

## Conclusions

SiNW arrays were successfully fabricated by the electroless etching of an n-Si (100) wafer in HF/AgNO_3_. The fabrication method is a simple, one-step, low-cost process that does not involve complicated equipment or procedures. The size (diameter) of the SiNWs ranges from 20 to 200 nm. Various shapes of SiNWs were found, including round, rectangular, and triangular shapes. The shape variation of SiNWs is suggested to be due to the inhomogeneous nucleation of silver on the Si substrate surface. A low reflectance of less than 10% was obtained in the near UV region to the near IR region. Thus, it was proven that the SiNWs can be used for antireflection applications over a broad spectral range. The band gap energy of the SiNWs is slightly higher than that of the Si substrate. Contact-mode AFM using a gold-coated tip was successfully applied for the electrical measurement of the SiNWs. The *I–V* characteristics of a freestanding SiNW show linear ohmic behavior. The average resistivity of a SiNW is approximately 33.94 Ω cm.

## Abbreviations

AFM: Atomic force microscopy
AgNO_3_: Silver nitrate
EDX: Energy dispersive X-ray
FESEM: Field-emission scanning electron microscopy
HCl: Hydrochloric acid
HF: Hydrofluoric acid
HNO_3_: Nitric acid
*I*–*V*: Current–voltage
SiNWs: Silicon nanowires
TEM: Transmission electron microscopy
